# Tissue prolapse-induced acute stent thrombosis: a case report and intravascular imaging-based insight

**DOI:** 10.3389/fcvm.2025.1637979

**Published:** 2025-07-10

**Authors:** Weifeng Zhang, Shengqi Jia, Yanmin Wu, Haiyan Jia

**Affiliations:** ^1^ Department of Cardiology, Affiliated Hospital of Hebei University, Baoding, China; ^2^ Department of Cardiology, Tianjin Union Medical Center, the First Affiliated Hospital of Nankai University, Tianjin, China

**Keywords:** stent thrombosis, tissue prolapse, percutaneous coronary intervention, intravascular ultrasound (IVUS), acute myocardial infarction

## Abstract

**Background:**

Acute stent thrombosis (AST) is a rare but serious complication occurring within 24 h after percutaneous coronary intervention (PCI), typically caused by insufficient antiplatelet therapy, stent under-expansion, or malapposition. Tissue prolapse (TP) within the stent is less frequently reported as a cause of AST and lacks direct imaging-based evidence.

**Case summary:**

We report a case of a patient with acute ST-segment elevation myocardial infarction (STEMI) who experienced recurrent ST-segment elevation 1 h after primary PCI. Coronary angiography revealed mid-stent occlusion, and intravascular ultrasound (IVUS) identified tissue prolapse with echogenic features consistent with fibrolipidic plaque. The initial stent was appropriately sized and well-deployed, and activated clotting time (ACT) indicated adequate anticoagulation. In the absence of traditional risk factors for AST, the large-volume tissue prolapse was considered the likely cause. Balloon angioplasty failed to resolve the prolapse, so an additional stent was implanted at the site. After this intervention, no further thrombotic events occurred.

**Conclusion:**

This case provides clear intravascular imaging evidence that tissue prolapse can induce AST. For cases of early stent thrombosis with angiographic haziness and no signs of dissection or malapposition, clinicians should suspect tissue prolapse. Intravascular imaging facilitates accurate diagnosis and helps guide treatment. Further research is warranted to define high-risk features of tissue prolapse—such as volume, protrusion, and composition—to establish thresholds and interventional criteria that may prevent AST.

## 1 Introduction

**Figure 1:**
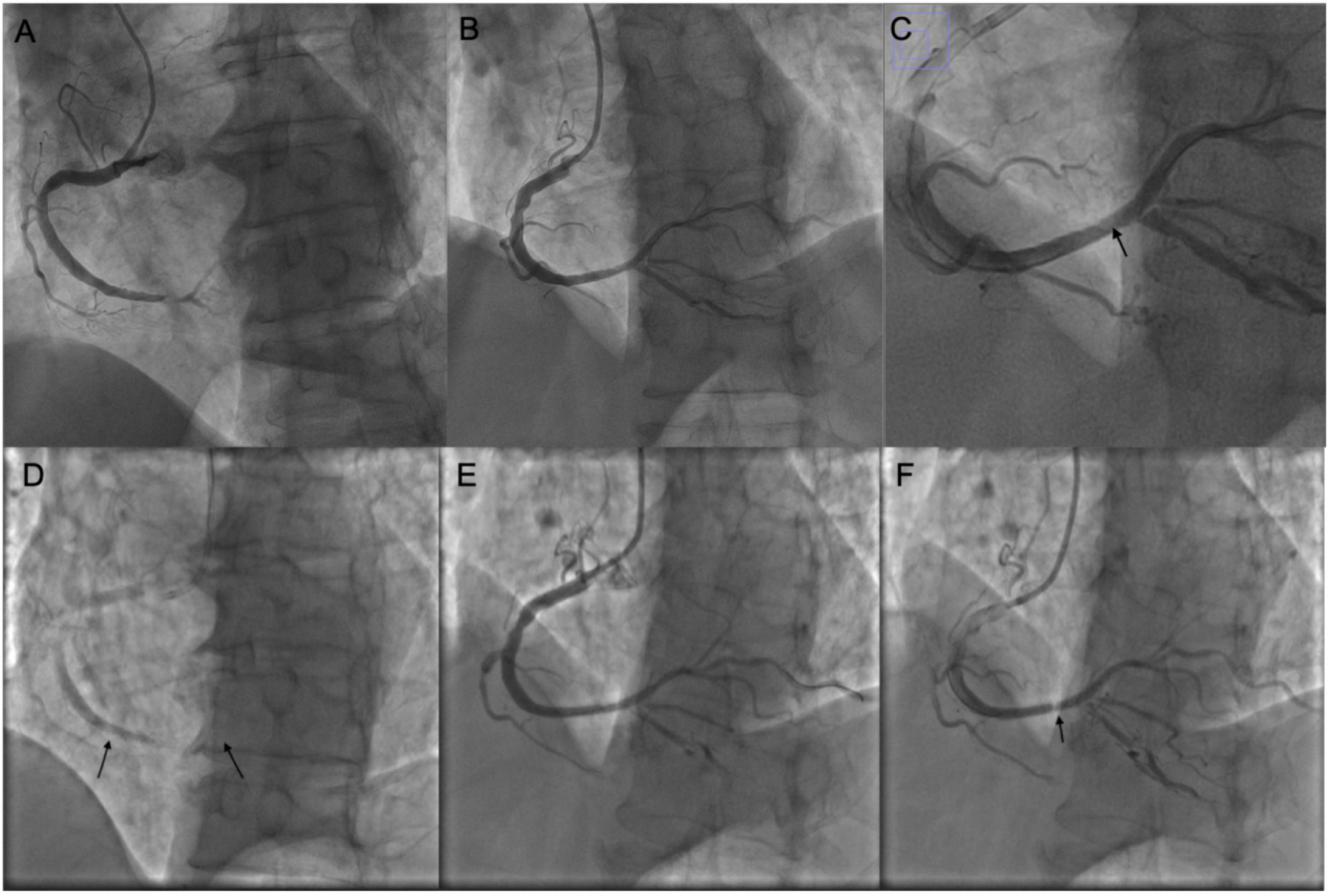
Coronary intervention images of the patient. **(A)** Coronary angiography showing subtotal occlusion of the posterolateral branch. **(B)** After the first PCI procedure. **(C)** Magnified image of panel **(B)**; the arrow indicates a pale area within the stent. **(D)** Before the second PCI; arrows indicate the proximal and distal edges of the stent. **(E)** After balloon dilatation during the second PCI; no dissection is observed at the stent edges. **(F)** Before concluding the second PCI, the pale area within the stent is noted again.

Case Details: A 73-year-old male patient was admitted with a chief complaint of chest pain for 2 h.
History of Present Illness: The patient experienced sudden onset chest pain 2 h prior to admission without any obvious triggers. The pain was accompanied by marked radiation, nausea, vomiting (gastric contents), and profuse sweating. Symptoms were persistent and unrelieved, prompting him to seek emergency care at the Emergency Department of the Affiliated Hospital of Hebei University. Electrocardiogram (ECG) revealed ST-segment elevation in the inferior leads, suggesting acute inferior wall myocardial infarction (MI). He was given aspirin 300 mg, ticagrelor 180 mg, and heparin 5,000 IU intravenously, after which his symptoms were relieved. The patient was then transferred to the Chest Pain Center for emergency PCI.
Past Medical History: The patient had a 5-year history of type 2 diabetes mellitus, managed with metformin (one tablet twice daily) and glipizide (one tablet each morning), with suboptimal glycemic control. He had a 40-year history of smoking (\∼30 cigarettes/day), and occasional alcohol consumption.
Physical Examination: T: 36.2°C, P: 40 bpm, R: 19 bpm, BP: 91/58 mmHg. The patient was conscious and cooperative. No cyanosis of the lips, clear lung sounds without rales, normal heart borders, muffled heart sounds, HR: 40 bpm, regular rhythm, no audible murmurs. Abdomen was soft, non-tender, with no rebound pain or guarding; liver and spleen not palpable; no edema in the lower limbs.
Auxiliary Tests: ECG: Third-degree AV block with junctional escape rhythm; ST-segment elevation in leads II, III, and aVF with a domed pattern.
  Echocardiography: Mildly dilated ascending aorta; degenerative changes with mild regurgitation in the aortic valve; posterior mitral leaflet calcification with mild regurgitation; mild tricuspid regurgitation; LVEDD 4.8 cm; LVEF 58%.
  CBC: WBC 10.43 × 10⁹/L, monocytes 0.63 × 10⁹/L, neutrophils 6.90 × 10⁹/L.
  cTnI: >50 ng/ml.
  BNP and D-dimer: within normal limits.
Preliminary Diagnosis: 1. Acute inferior wall myocardial infarction, Killip class I.
  Arrhythmia: third-degree AV block with junctional escape rhythm2. Type 2 diabetes mellitus.
Differential Diagnosis:
  1. Aortic dissection: Typically presents with abrupt, severe chest pain radiating to the abdomen and limbs, often with hypertension and blood pressure discrepancy between arms. Not supported in this case.
  2. Acute pulmonary embolism: Often associated with prolonged immobilization, DVT, and signs of hypoxemia such as dyspnea and cyanosis. Not supported in this case.
Primary PCI: Coronary angiography showed: Right-dominant coronary circulation. LM: plaques; LAD: plaques with 80% stenosis in the proximal-mid segment, 90% stenosis at D2 ostium; LCX: small-caliber vessel with 70% proximal stenosis; RCA: plaques with 95% stenosis in the proximal segment before the PDA; TIMI flow grade 2.
Intervention:
  Intra-procedural ACT: 300 s
  After PTCA, a 3.0 × 36 mm drug-eluting stent (Xience) was deployed in the RCA (inflation: 14 atm, 20 s).
  Post-dilation with NC Dmax 3.5 × 12 mm balloon (16 atm).
  Post-intervention TIMI flow: grade 3.
  No evidence of edge dissection (Figure 1).
  Blood pressure and heart rate returned to normal post-op.
Acute In-Stent Thrombosis Event:

Approximately 60 min post-op, the patient experienced recurrent chest pain. ECG again showed ST-segment elevation in inferior leads. Urgent repeat PCI was performed. Angiography revealed acute stent thrombosis with total occlusion in the RCA. Intra-procedural ACT: 290 s Balloon dilatation with 2.0 × 15 and 3.0 × 12 mm balloons restored flow. No edge dissection observed.

Investigation of Cause:

*Initial suspicion* was underexpansion or malapposition of the stent. However, considering:

Absence of significant RCA calcification,
Appropriate stent size and deployment pressure/duration,
Adequate post-dilation with a 3.5 mm balloon,
Malapposition or underexpansion was unlikely.

**Figure 2:**
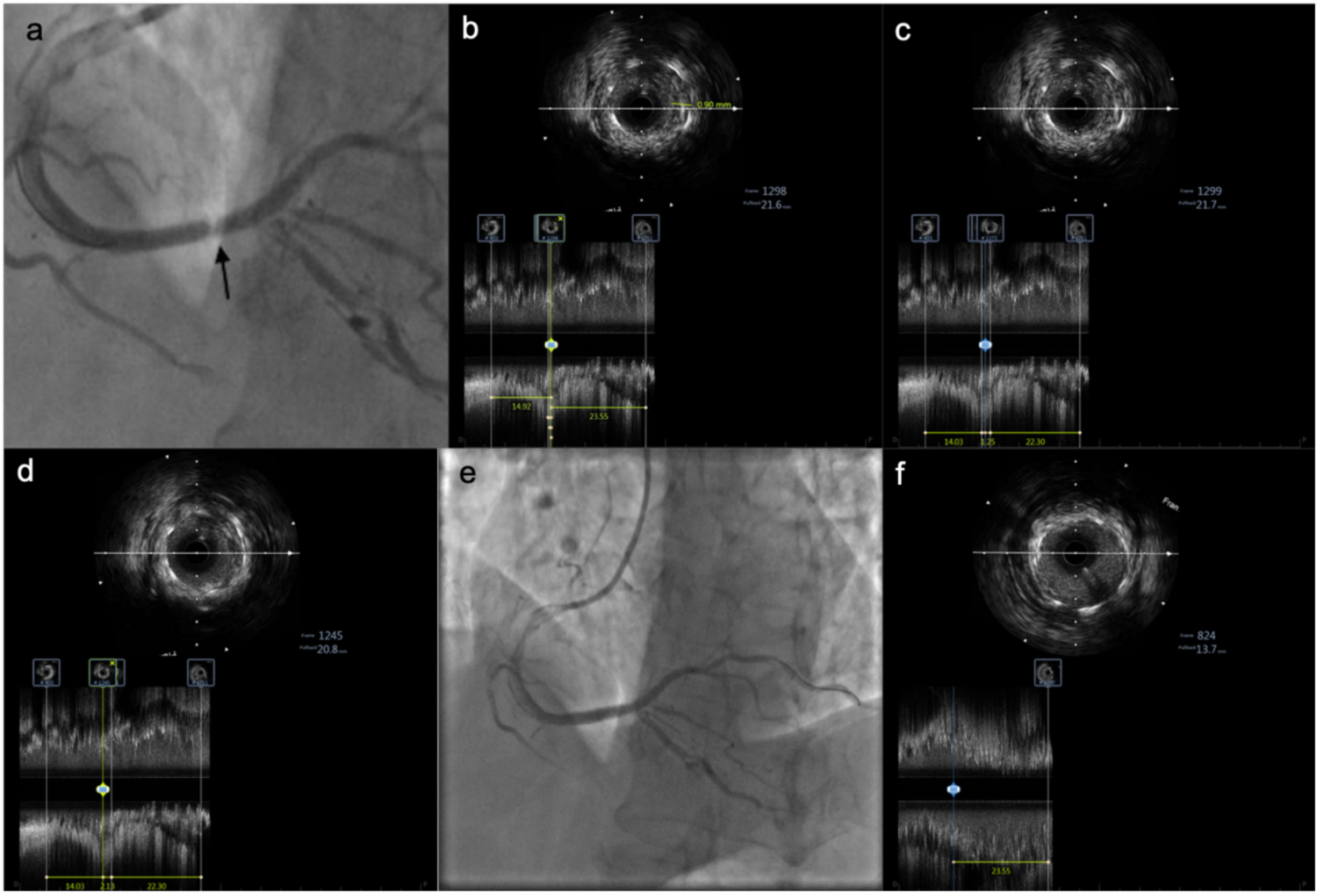
IVUS images showing tissue prolapse within the stent. **(a)** Pale appearance within the stent. **(b)** IVUS examination reveals tissue prolapse with a thickness of 0.9 mm. **(c)** Irregular tissue prolapse with an estimated area of approximately 2.01 mm^2^. **(d)** Length of the prolapsed tissue is approximately 2.13 mm. **(e)** Angiographic image after implantation of an additional stent at the site of prolapse. **(f)** At the same proximal stent location, mild tissue prolapse is observed at the 12 o'clock position, significantly improved compared to before.

*IVUS was performed* for further clarification and revealed: Tissue prolapse in the mid-portion of the stent, with:

Thickness: 0.9 mm,
Length: 2.13 mm,
Area: \∼2.01 mm^2^ (Figure 2).
No edge dissection; good stent expansion and apposition; no stent fracture.
*Repeat angiography revealed* focal “hazy” appearance in mid-stent, consistent with tissue prolapse. Further balloon dilation with NC Senor 3.0 × 15 mm (14 atm, 30 s) partially improved haziness. Repeat IVUS confirmed persistent tissue prolapse.
After several minutes, angiography again showed worsening of haziness, confirming acute stent thrombosis secondary to tissue prolapse, refractory to balloon dilation.

Final Treatment:
  A 3.5 × 16 mm drug-eluting stent (Boston Scientific Promus Premier) was implanted at 12 atm for 20 s.
  Post-dilation with NC Dmax 3.5 × 12 mm high-pressure balloon.
  Final angiography showed excellent stent apposition, no dissection or rupture, TIMI 3 flow.
  IVUS showed significantly reduced prolapsed tissue area, no edge dissection, and well-expanded stent.
Outcome:

The patient did not experience recurrent chest pain or further thrombotic events post-procedure.

## 2 Discussion

### 2.1 Mechanisms and risk factors of acute stent thrombosis (AST)

Acute stent thrombosis (AST) refers to an acute occlusion event due to thrombus formation within the stent within 24 h after percutaneous coronary intervention (PCI). Clinically, it often presents with ST-segment re-elevation, malignant arrhythmias, or cardiogenic shock. The incidence of AST is approximately 0.2%–0.6% (1). Drug-related factors include inadequate anticoagulation or antiplatelet therapy. Lesion- and procedure-related causes primarily include stent underexpansion, poor stent apposition, and edge dissection. Tissue prolapse within the stent is a less frequently discussed cause of AST.

### 2.2 Tissue prolapse within the stent: definition, incidence, and clinical significance

Tissue prolapse (TP) refers to a phenomenon in which plaque components or other tissue material protrude into the stent lumen through the stent struts after deployment, typically visualized by optical coherence tomography (OCT) or intravascular ultrasound (IVUS). TP is not uncommon. A subgroup analysis of the ADAPT-DES (Assessment of Dual Antiplatelet Therapy with Drug-Eluting Stents) study (2) found that 34.3% of patients had tissue prolapse on IVUS after stent implantation. However, this study indicated that TP was not associated with worse long-term outcomes, possibly because lesions with TP had better stent expansion. In contrast, another study (3) suggested that TP was associated with adverse short-term events (e.g., acute and subacute stent thrombosis, no-reflow phenomenon) but not with poor long-term prognosis. Notably, the volume of tissue prolapse in 14 patients with acute/subacute stent thrombosis was significantly greater than that in 128 TP patients without thrombosis (3.3 ± 1.6 vs. 2.6 ± 1.9 mm^3^, *p* = 0.012). Another OCT-based study reported that tissue prolapse thickness >500 μm was associated with adverse outcomes (3). Furthermore, the JSCAI (Journal of the Society for Cardiovascular Angiography & Interventions) expert consensus also identified plaque or thrombus prolapse as a potential contributor to acute stent thrombosis (1).

### 2.3 Case characteristics: imaging evidence of Tp-induced AST

This patient presented with acute inferior ST-elevation myocardial infarction (STEMI). After initial PCI, ST-segment elevation recurred within 60 min, and repeat angiography showed mid-stent occlusion at the same lesion site. No signs of stent edge dissection were observed after balloon dilation, confirming the diagnosis of acute stent thrombosis (AST). Although AST occurred, the thrombus burden appeared low on angiography after balloon dilation. Moreover, according to the 2025 ACC/AHA/ACEP/NAEMSP/SCAI Guideline for the Management of Patients With Acute Coronary Syndromes, routine thrombus aspiration is not recommended in primary PCI. Therefore, thrombus aspiration was not performed.

Initially, poor stent expansion or malapposition was suspected. However, intraprocedural assessment estimated the reference vessel diameter to be 3.25 mm proximally and 2.75 mm distally, and a 3.0-mm stent was deployed at 14 atm for 20 s, with no signs of underexpansion. Post-dilatation with a 3.5-mm balloon at a maximum pressure of 24 atm was performed. Therefore, underexpansion and malapposition were unlikely causes.

In terms of pharmacologic therapy, the patient's activated clotting time (ACT) was 300 s during the first PCI, indicating sufficient anticoagulation. Moreover, aspirin and a loading dose of ticagrelor had been administered, ensuring adequate antiplatelet therapy. Thus, inadequate antithrombotic therapy was also ruled out.

Reviewing angiographic images after the first PCI, a pale appearance was noted at the original lesion site within the stent. Subsequent IVUS imaging revealed tissue prolapse in this region, with a maximum area of 2.01 mm^2^ and length of 2.13 mm. An initial strategy of repeat balloon dilation was chosen, but 5 min after dilation, the pale appearance worsened on angiography, and IVUS still showed no improvement in the prolapse. The ACT remained at 290 s, confirming continued adequate anticoagulation. This strongly suggested that AST was related to the tissue prolapse.

Although the use of drug-coated balloons (DCB) was considered, it was not pursued because DCBs are primarily indicated for in-stent restenosis and small vessel disease. This case involved acute in-stent thrombosis, which falls outside the typical indications for DCB use. Moreover, as the underlying cause was tissue prolapse rather than neointimal hyperplasia, DCB treatment would not have resolved the mechanical issue. The worsening of the hazy appearance shortly after balloon dilation further indicated that balloon angioplasty alone was insufficient.

To prevent recurrent thrombosis, a second stent was implanted at the prolapse site. Post-stent imaging showed significant improvement in the prolapse, and no further thrombotic events occurred.

### 2.4 Clinical implications and future perspectives

Based on previous literature (2–5), this case provides the following insights:

1. Although the clinical impact of TP after PCI remains controversial, current guidelines and expert consensus (1) recognize its association with stent thrombosis. The risk appears to depend on the volume of prolapse (3, 5). In this case, the prolapse measured 2.01 mm^2^ in area and 0.9 mm in thickness, supporting this notion.
2. A pale appearance within the stent on angiography may be due to thrombus, TP, or other causes. In cases of unexplained AST, IVUS can help clarify the underlying pathology.
3. Management options for TP include balloon dilation; however, post-dilation imaging is essential to assess changes in the stent appearance. In this case, balloon dilation was ineffective, and additional stent implantation was required to prevent further thrombosis.
4. Further studies are needed to quantify high-risk features of TP—such as area, protrusion extent, and tissue characteristics—and to establish thresholds and interventional criteria to guide intra-procedural decision-making and reduce the incidence of AST.

## Data Availability

The original contributions presented in the study are included in the article/Supplementary Material, further inquiries can be directed to the corresponding author.
